# Parental knowledge, attitudes, and discretionary practices regarding children’s dietary salt intake in Saudi Arabia: a cross-sectional study

**DOI:** 10.3389/fpubh.2026.1894899

**Published:** 2026-09-28

**Authors:** Almoutaz Hashim, Enad Alsolami, Sami Alobaidi

**Affiliations:** Department of Internal Medicine, University of Jeddah, Jeddah, Saudi Arabia

**Keywords:** children, knowledge, attitudes, and practices, parents, salt intake, Saudi Arabia, sodium reduction

## Abstract

**Objectives:**

To assess salt-related knowledge, attitudes, and practices (KAP) among parents and caregivers in Saudi Arabia and examine associations between parental awareness and child-directed salt-reduction behaviors.

**Methods:**

This cross-sectional analysis included 655 parents or caregivers of children under 18 years of age from a broader online convenience sample surveyed between December 2022 and May 2023. A 37-item questionnaire adapted from established instruments and translated into Arabic using a forward-backward process assessed salt-related KAP. Analyses included descriptive statistics, exploratory chi-square tests with Cramér’s V, and multivariable binomial logistic regression. Benjamini–Hochberg false-discovery-rate (FDR) correction was applied across the 16 primary KAP regression coefficients.

**Results:**

Among participants, 78.8% recognized excessive salt intake among Saudi adults, compared with 54.2% for Saudi children, and 95.7% recognized the general health risks of high salt intake. Most (89.5%) considered limiting their child’s salt intake important, yet 82.6% reported adding salt to foods prepared for their children. After multivariable adjustment and FDR correction, support for more action to reduce salt in foods targeted at children was independently associated with not placing a salt shaker on the table at mealtimes (aOR = 3.80, 95% CI 1.65–8.79, raw *p* = 0.002, *q* = 0.032); the wide confidence interval indicates limited precision. The inverse association between recognizing excessive salt intake among Saudi children and requesting meals prepared without salt when dining out (raw *p* = 0.035) did not remain significant after FDR correction (*q* = 0.280). Other adjusted associations were not significant, and unadjusted chi-square findings were considered exploratory.

**Conclusion:**

Parents and caregivers in Saudi Arabia showed high general awareness of salt-related health risks but lower recognition of excessive salt intake among children. The findings also suggest a knowledge–behavior gap, with frequent discretionary salt use despite favorable attitudes toward limiting children’s salt intake. Adjusted analyses provided limited evidence of independent associations between parental KAP and selected behaviors, with only one association surviving FDR correction. These findings support culturally appropriate, structurally supportive sodium-reduction strategies for children and families in Saudi Arabia.

## Introduction

Excessive dietary salt consumption is an important health concern worldwide (1). Globally, both adults and children frequently consume salt at levels that far exceed the recommendations set by the World Health Organization (WHO) (1). This chronic exposure to elevated salt levels is strongly linked to multiple adverse health outcomes, mainly elevated blood pressure and an increased risk of cardiovascular disease (2). The impact of chronic excess salt intake is not limited to adults; early life dietary habits can also predispose children to long-term negative health outcomes, setting the stage for chronic diseases later in life (3). Considering the important role of early dietary practices in shaping lifelong health trajectories, it is essential to examine the multiple factors that influence children’s salt consumption (4). These factors encompass not only individual behavioral choices but also parental influences and other socio-cultural practices (4). Therefore, well-conducted studies exploring the factors that influence children’s dietary salt intake across different cultural contexts are essential for developing targeted community interventions to reduce salt intake and mitigate associated health risks over time. Moreover, the WHO originally set a target to reduce population salt intake by 30% by 2025 (5). Achieving this goal also requires comprehensive strategies informed by an understanding of consumer behavior. Parents play a pivotal role in shaping children’s eating habits (6). Therefore, assessing their knowledge, attitudes, and practices related to salt intake is essential for designing effective interventions.

High consumption of salt-rich foods among young Saudis highlights the need to better understand factors influencing children’s dietary salt intake in Saudi Arabia (7). While research in neighboring countries such as Oman has shown links between maternal nutritional awareness and children’s dietary intake (8), Saudi data on salt-related knowledge and practices remain limited, particularly among parents of children (9). The unique cultural, social, and dietary patterns of Saudi families warrant specific investigation, as local eating habits and food preparation methods may significantly influence children’s salt consumption.

Currently, there are limited data on Saudi parents’ knowledge and attitudes regarding children’s salt intake. This study aims to bridge this gap by assessing salt-related KAP among Saudi parents, providing valuable insights for future health initiatives.

This research is particularly relevant given Saudi Arabia’s rapid dietary transition, including increased consumption of processed and fast foods, which can contribute substantially to sodium intake. By identifying knowledge gaps and attitudinal barriers among Saudi parents, healthcare providers and policymakers can design culturally appropriate interventions to promote healthier dietary practices and reduce excessive salt intake among children in the Kingdom (10).

## Materials and methods

### Study design, recruitment, and sampling considerations

This investigation is part of a larger cross-sectional study designed to evaluate the knowledge, attitudes, and behaviors related to dietary salt intake among the Saudi population (11). The primary study was conducted from December 2022 to May 2023 utilizing an online survey hosted via Google Forms, targeting Saudi residents aged 18–65 years. The project was approved by the institutional ethical committee/institutional review board, approval number UJ-REC-299, and this single, uniform approval covers all elements of data collection reported herein.

Within the larger study, participants—comprising both Saudi nationals and non-Saudi residents—were recruited through online platforms, community outreach initiatives, and digital networks. The present study focuses specifically on a sub-cohort of this data: individuals who explicitly identified as parents or primary caregivers of at least one child under the age of 18 years. Out of the broader sample, a final subset of 655 parents or caregivers completed all child-specific metrics and were included in this analysis.

### Survey development and validation

The study utilized a 37-item questionnaire to assess knowledge, attitudes, and practices (KAP) regarding dietary salt intake. The instrument comprised three main sections: demographic characteristics (11 items), general salt-related KAP (18 items), and child-specific salt-related KAP (8 items).

The survey questions were adapted from established, previously validated international salt-assessment instruments (12). To support linguistic and cultural equivalence within the Saudi Arabian context, a structured translation protocol was conducted. First, a bilingual public health researcher translated the original English instrument into Arabic. Second, a separate independent expert, blinded to the original English text, performed a back-translation into English. Both versions were compared to resolve structural or idiomatic discrepancies. Following translation, the draft Arabic survey was evaluated by an expert review panel consisting of three experts to assess face and content validity in relation to local dietary habits. A pilot test was subsequently conducted with 20 Saudi adults from diverse educational backgrounds to optimize item clarity, layout, and readability, and data from this pilot group were excluded from the final analysis. Because the survey items primarily comprised separate categorical knowledge, attitude, and behavior items rather than a continuous psychometric scale, formal statistical reliability testing, such as Cronbach’s alpha, was not performed.

Demographic information gathered encompassed age, gender, nationality, education, occupation, monthly income, and marital status. In addition, participants reported on their smoking behaviors and levels of physical activity. We also collected details regarding any history of hypertension or other chronic illnesses, along with the use of antihypertensive drugs.

### Study focus and question selection

This analysis centers on eight specific questions answered by parents/caregivers of children under 18 years, alongside eight selected questions from the broader adult KAP assessment. The selection included two knowledge-based questions and four behavior-related questions, chosen based on previous research indicating associations between specific knowledge and salt-related behaviors. Two additional attitude questions were included to understand parents’ perceptions of salt intake relative to other nutrients.

### Parental knowledge assessment

The evaluation of parents’ knowledge regarding children’s salt intake involved two key questions using five-point Likert scales:

Perception of salt consumption among Saudi children (ranging from “far too much” to “far too little”).

Understanding of long-term health impacts of excessive salt intake during childhood (ranging from “strongly disagree” to “strongly agree”).

### Parental knowledge of adult salt intake

One question evaluated parents’ perception of salt consumption among the general Saudi population by asking, “In general, how much salt do you think people in Saudi Arabia eat?” with options ranging from “far too much” to “far too little” or “do not know.” A second question gauged their awareness of the health risks associated with excessive salt intake, asking, “Do you think that eating too much salt could damage your health?” with possible responses of “yes,” “no,” or “do not know.”

### Parental attitudes on children’s salt intake

Two five-point Likert scale questions were used to assess parental attitudes regarding children’s salt consumption. Respondents rated their agreement with the statements “Limiting the amount of salt my child(ren) eat(s) is important to me” and “More action needs to be taken to reduce salt in foods targeted at children,” with options ranging from “strongly disagree” to “strongly agree.”

### Parental attitudes on adult salt intake

Parents’ attitudes toward adult salt consumption were measured with two questions. One question asked them to compare their own daily salt intake with health recommendations, offering choices such as “I eat less salt than recommended,” “I eat about the right amount,” “I eat more salt than recommended,” or “I do not know.” The second question evaluated their level of concern about six food-related issues (healthy eating, sugar, salt, fat, saturated fat, and calories) using a five-point scale from “not at all concerned” to “extremely concerned.”

### Salt-related behaviors

For children, three questions evaluated parents’ behaviors regarding discretionary salt use: adding salt during food preparation, placing a salt shaker on the table at meals, and the frequency of children adding salt at the table. For their own salt use, parents answered three similar questions on adding salt during cooking, at the table, or keeping a salt shaker available. Additionally, parents reported the frequency of various salt-reduction practices over the past month—such as reading food labels for sodium content, purchasing low-salt products, avoiding packaged or fast foods, using herbs instead of salt in cooking, and requesting low-salt meals when dining out—with response options ranging from “always” to “never,” including “does not apply to me.”

### Statistical analysis

Data analysis was conducted using Jamovi (The Jamovi Project, 2025; Version 2.6) and Python 3 with the statsmodels package. Descriptive statistics were calculated to summarize the socio-demographic characteristics of the participants. Chi-square tests were performed to assess whether there were significant differences in categorical responses, with effect sizes reported using Cramér’s V to provide additional context for exploratory findings. All statistical tests were two-tailed. For the exploratory chi-square analyses, nominal *p*-values are reported, whereas statistical significance for the 16 primary KAP regression coefficients was assessed after Benjamini–Hochberg false-discovery-rate correction using *q* < 0.05.

Four separate multivariable binomial logistic regression models were fitted, one for each binary behavioral outcome. In each model, the four knowledge/attitude predictors were entered simultaneously and adjusted for parental age, sex, educational attainment, monthly household income, smoking status, physical activity, and chronic disease history. Thus, each KAP coefficient is mutually adjusted for the other three KAP predictors and for the listed covariates. Adjusted odds ratios (aORs), 95% confidence intervals (CIs), and two-sided *p*-values were reported. To account for multiple testing across the 16 primary KAP coefficients, Benjamini–Hochberg false-discovery-rate (FDR) corrections were applied, generating *q*-values where a threshold of *q* < 0.05 determined statistical significance after multiplicity correction.

## Results

### Demographic characteristics

A total of 655 parents and caregivers, each with at least one child under the age of 18, completed the online survey. The majority (70.5%) of respondents were female (Table 1), with a mean age of 40.4 years (SD = 8.02). Most participants were Saudi nationals (88.2%), had bachelor’s/diploma or postgraduate education (77.1%), and were married (89.5%). Selected demographic details are summarized in Table 1.

**Table 1:** Demographic characteristics of participants.

Variables	*n* (%)
Gender	
Male	193 (29.5%)
Female	462 (70.5%)
Nationality	
Saudi	578 (88.2%)
Non-Saudi	77 (11.8%)
Education	
High school or less	150 (22.9%)
Bachelor’s/Diploma	418 (63.8%)
Master’s or PhD	87 (13.3%)
Occupation	
Unemployed	358 (54.7%)
Government Sector	171 (26.1%)
Military Sector	22 (3.4%)
Private Sector	104 (15.9%)
Monthly income (in Saudi Riyals)	
<5,000	259 (39.5%)
5,000–15,000	227 (34.7%)
>15,000	169 (25.8%)
Marital status	
Married	586 (89.5%)
Divorced/single/separated/widowed	69 (10.5%)
Physical activity	
No regular physical activity	345 (52.7%)
Physical activity < 150 min/week	220 (33.6%)
Physical activity > 150 min/week	90 (13.7%)

### Knowledge

Overall, 78.8% of parents recognized that Saudi adults consume excessive amounts of salt, while a smaller proportion (54.2%) acknowledged that children in Saudi Arabia do the same. A significant majority (95.7%) understood that high salt intake can be detrimental to health; however, 91.0% agreed that excessive salt intake during childhood may have harmful long-term effects on children’s health.

### Attitudes

Just over half (59.4%) of the parents believed their own salt intake was at or below the recommended daily level, while only 16.8% felt their intake exceeded these recommendations. Approximately 34.6% expressed high concern about their dietary salt intake. In contrast, more parents reported being very or extremely concerned about sugar intake (39.7%), saturated fat (46.3%), healthy eating practices (34.8%), and overall fat consumption (44.2%). Fewer parents (22.9%) were concerned about calorie intake. Regarding their children’s diets, the vast majority (89.5%) agreed that it was important to limit their child’s salt consumption, and an even larger proportion (93.9%) felt that more action should be taken to reduce salt in foods targeted at children. Parents’ knowledge and attitudes towards salt intake related to their child/children are summarized in Table 2.

**Table 2:** Parents’ knowledge and attitudes towards salt intake related to their child/children.

Statements	*n* (%)
Part A: Child-targeted attitudes & knowledge	
In the long term eating too much salt during childhood may have harmful effects on children’s health	
Strongly Disagree	0
Disagree	6 (0.9%)
Neither agree nor disagree	53 (8.1%)
Agree	245 (37.4%)
Strongly agree	351 (53.6%)
Limiting the amount of salt my child/or children eat/s is important to me	
Strongly Disagree	3 (0.5%)
Disagree	4 (0.6%)
Neither agree nor disagree	62 (9.5%)
Agree	251 (38.3%)
Strongly agree	335 (51.1%)
Part B: Structural/policy-level attitudes	
More action needs to be taken to reduce the salt in foods targeted at children	
Strongly Disagree	1 (0.2%)
Disagree	1 (0.2%)
Neither agree nor disagree	38 (5.8%)
Agree	169 (25.8%)
Strongly agree	446 (68.1%)

### Behaviors

Approximately 41.4% of parents reported adding salt to their own food at the table, while less than one-fourth (18%) indicated that their child added salt to food at mealtimes. Most parents (93.9%) added salt during cooking, but fewer (82.6%) reported doing so for foods prepared for their children. Additionally, 14.7% of parents placed a salt shaker on the table during meals.

In terms of actions taken to reduce dietary salt in the past month, common behaviors included avoiding Asian-style restaurants or takeaway stores (41.1%), packaged ready-to-eat foods (37.7%), and fast food restaurants (36.3%). Other behaviors included purchasing foods labeled as having no added or reduced salt/sodium (22.4%) and using spices and herbs instead of salt in cooking (18.6%). Fewer parents consistently checked food labels for sodium/salt content (11.6%) or requested meals prepared without salt when dining out (5%). Selected parent and child-related discretionary salt use behaviors are summarized in Table 3.

**Table 3:** Discretionary salt use behaviors of parents and those related to their child/children.

Responses	*n* (%)
Part A: Child-targeted discretionary behaviors	
Do you add salt to foods you prepare for your child or children?	
Always	223 (34.0%)
Often	184 (28.1%)
Sometimes	134 (20.5%)
Rarely	51 (7.8%)
Never	41 (6.3%)
Not applicable	22 (3.4%)
Does your child or children add salt to their food at the table?	
Always	20 (3.1%)
Often	22 (3.4%)
Sometimes	76 (11.6%)
Rarely	92 (14.0%)
Never	444 (67.8%)
Do not know	1 (0.2%)
Part B: Caregiver self-related and home environment items	
Do you place a salt shaker on your table at meal times?	
Always	30 (4.6%)
Often	19 (2.9%)
Sometimes	47 (7.2%)
Rarely	50 (7.6%)
Never	509 (77.7%)

### Association of salt-related knowledge and attitudes with behaviors

The unadjusted chi-square analyses detailed below are interpreted as exploratory. Parents who recognized that children in Saudi Arabia consume too much salt differed in reported discretionary salt use when preparing food for their children (χ^2^ = 57.8, df = 25, *p* < 0.001, Cramér’s V = 0.133) (Figure 1). There was no significant difference in discretionary salt use regarding children’s use of salt at the table or placing a salt shaker on the table during meals.

**Figure 1:**
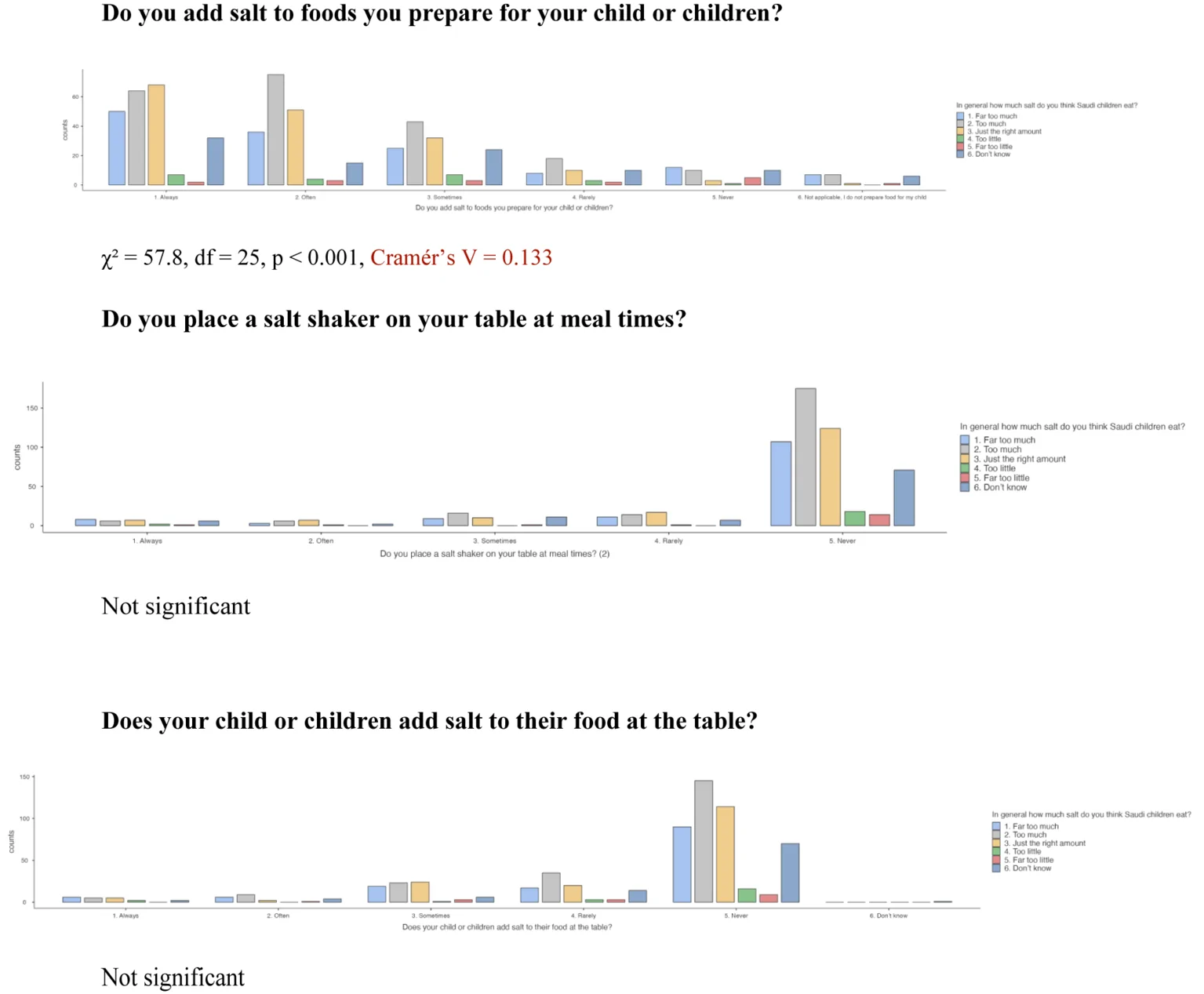
Association between discretionary salt use behaviors of parents and those related to their child/children and parents’ knowledge that Saudi children eat too much salt.

Those aware that children in Saudi Arabia eat too much salt were more inclined to check food labels for sodium/salt content (χ^2^ = 38.7, df = 25, *p* = 0.039, Cramér’s V = 0.109) and to request meals prepared without salt when dining out (χ^2^ = 48.0, df = 25, *p* = 0.004, Cramér’s V = 0.121).

Parents who recognized that Saudi adults consume excessive amounts of salt did not show significant associations with discretionary salt use behavior; however, they were more likely to use spices and herbs as alternatives to salt in cooking (χ^2^ = 52.8, df = 25, *p* < 0.001, Cramér’s V = 0.127) and request meals prepared without salt when eating out (χ^2^ = 42.3, df = 25, *p* = 0.017, Cramér’s V = 0.114).

Parents who agreed that excessive salt intake during childhood could have harmful long-term effects on health were more likely to add salt to food prepared for their child (χ^2^ = 25.9, df = 15, *p* = 0.039, Cramér’s V = 0.115) and less likely to place a salt shaker on the table during meals (χ^2^ = 23.6, df = 12, *p* = 0.023, Cramér’s V = 0.110) (Figure 2). Additionally, they were more likely to avoid fast food restaurants (χ^2^ = 54.5, df = 15, *p* < 0.001, Cramér’s V = 0.167), purchase foods labeled as having no added or reduced salt/sodium (χ^2^ = 45.3, df = 15, *p* < 0.001, Cramér’s V = 0.152), and request meals prepared without salt when dining out (χ^2^ = 25.4, df = 15, *p* = 0.045, Cramér’s V = 0.114).

**Figure 2:**
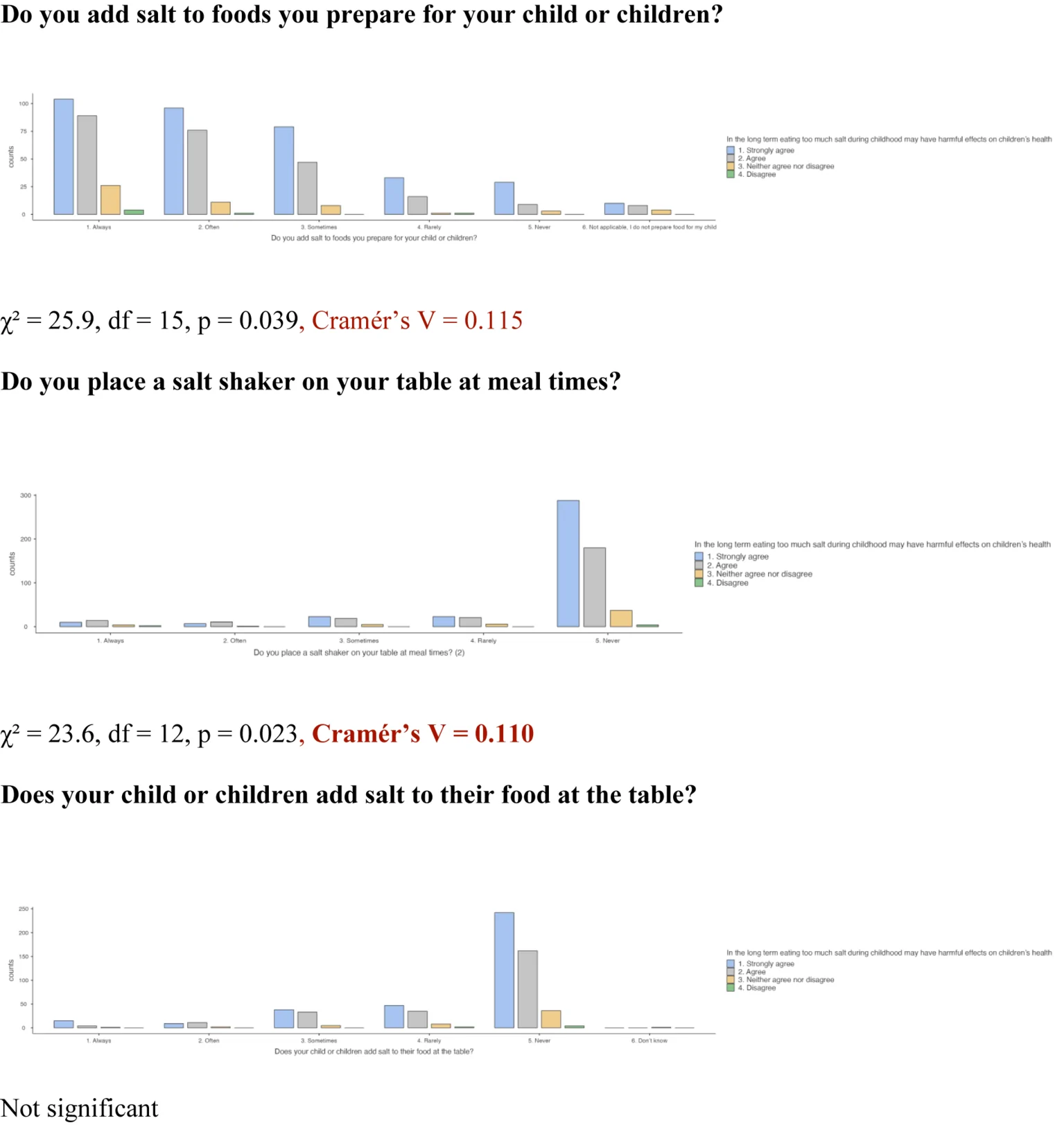
Association between parents’ knowledge of the long-term health implications of eating too much salt during childhood and discretionary salt use behaviors related to their child/children.

Parents who acknowledged the importance of limiting their child’s salt intake were less likely to place a salt shaker on the table during meals (χ^2^ = 31.8, df = 16, *p* = 0.011, Cramér’s V = 0.110) and reported that their children added salt to food at the table less frequently (χ^2^ = 227.0, df = 20, *p* < 0.001, Cramér’s V = 0.294). They were also more likely to avoid packaged ready-to-eat foods (χ^2^ = 35.5, df = 20, *p* = 0.017, Cramér’s V = 0.116), fast food restaurants (χ^2^ = 35.6, df = 20, *p* = 0.017, Cramér’s V = 0.117), purchase foods labeled as having no added or reduced salt/sodium (χ^2^ = 49.9, df = 20, *p* < 0.001, Cramér’s V = 0.138), and request meals prepared without salt when dining out (χ^2^ = 39.6, df = 20, *p* = 0.006, Cramér’s V = 0.123).

Four outcome-specific multivariable binomial logistic regression models were evaluated, one for each binary behavioral outcome. After Benjamini–Hochberg correction across the 16 primary KAP coefficients in Table 4, support for more action to reduce salt in foods targeted at children remained independently associated with not placing a salt shaker on the table at child mealtimes (aOR = 3.80, 95% CI 1.65–8.79; raw *p* = 0.002; q = 0.032). Conversely, the inverse association between perceiving that Saudi children consume too much salt and requesting a meal without salt when eating out (aOR = 0.57, 95% CI 0.34–0.96; raw *p* = 0.035) did not remain statistically significant after FDR correction (q = 0.280). Other mutually adjusted associations were not statistically significant (Table 4).

**Table 4:** Multivariable binomial logistic regression examining associations between parental knowledge/attitudes and salt-related behaviors.

Outcome	*N*	Predictor	aOR (95% CI)	Raw *p*	BH q
Checked food labels for salt/sodium content	631	Children eat too much salt	0.74 (0.51–1.08)	0.119	0.512
	631	Limiting child salt is important	0.89 (0.46–1.71)	0.717	0.983
	631	Childhood high salt harmful long-term	1.66 (0.76–3.63)	0.200	0.533
	631	More action needed for child-targeted foods	1.99 (0.78–5.07)	0.148	0.512
Asked for meal prepared without salt when eating out	609	Children eat too much salt	0.57 (0.34–0.96)	0.035	0.280
	609	Limiting child salt is important	2.22 (0.73–6.72)	0.160	0.512
	609	Childhood high salt harmful long-term	1.01 (0.32–3.21)	0.986	0.986
	609	More action needed for child-targeted foods	1.46 (0.45–4.73)	0.531	0.983
Did not place salt shaker on table at child mealtimes	655	Children eat too much salt	1.05 (0.63–1.73)	0.861	0.986
	655	Limiting child salt is important	1.15 (0.51–2.60)	0.737	0.983
	655	Childhood high salt harmful long-term	0.86 (0.35–2.10)	0.735	0.983
	655	More action needed for child-targeted foods	3.80 (1.65–8.79)	0.002	0.032
Did not add salt to food prepared for child	633	Children eat too much salt	0.88 (0.54–1.45)	0.627	0.983
	633	Limiting child salt is important	1.06 (0.44–2.58)	0.898	0.986
	633	Childhood high salt harmful long-term	1.80 (0.60–5.42)	0.293	0.670
	633	More action needed for child-targeted foods	1.03 (0.36–2.99)	0.954	0.986

aOR, adjusted odds ratio; CI, confidence interval; BH q, Benjamini–Hochberg false-discovery-rate-adjusted *p*-value across the 16 KAP coefficients. Four separate outcome-specific models were fitted. Each model simultaneously included all four knowledge/attitude predictors and adjusted for age, sex, educational attainment, monthly household income, smoking status, physical activity, and chronic disease history. Model-specific N varies because missing and “does not apply/not applicable” outcome responses were excluded by complete-case analysis. Number of children was unavailable. Statistical significance after multiplicity correction was assessed using *q* < 0.05.

## Discussion

This study represents an initial exploration of the relationship between salt-related knowledge and attitudes and behaviors among parents and caregivers in Saudi Arabia. Generally, parents demonstrated a relatively high level of understanding regarding the risks of excessive salt consumption, with the vast majority (95.7%) recognizing its potential harm to health. This finding is consistent with earlier research conducted in Saudi Arabia, in which nearly 95% of participants recognized that high salt intake could adversely affect health (9).

However, a slightly smaller proportion of parents (91.0%) agreed that excessive salt intake during childhood may have harmful long-term effects on children’s health. This awareness level is higher than that reported in an Australian parent study using comparable methods, in which 77% of parents were aware of the long-term risks associated with high salt intake in children (12). Another Australian parent-focused study similarly reported high recognition of salt-related health harms in children, although awareness of specific recommendations was lower (13). Most parents (78.8%) recognized that the Saudi population generally consumes too much salt, while fewer (54.2%) were aware that children also consume excessive amounts. While parents are largely aware of the health risks associated with high salt intake, these results suggest a gap in their understanding of children’s specific salt consumption levels. Culturally appropriate interventions may help address these gaps in knowledge and promote healthier dietary practices (14).

Our findings indicate that 89.5% of parents agreed that limiting salt in their child/children’s diet was important, whereas 34.6% reported high concern about the amount of salt in their own diet. Similar trends have been observed previously; for instance, parent-focused Australian research has reported support for reducing salt intake in children (15). While another study reported a lower level of parental concern (53%) regarding salt/sodium in children’s diets (12), our results, along with these previous studies, consistently reveal parental agreement on the need for increased efforts to reduce salt in foods marketed towards children (16). This suggests that parents may be receptive to initiatives aimed at reducing salt consumption in children (17).

Our study indicated that parents reported lower salt use by their children at the table compared with their own discretionary salt use. While 93.9% of parents reported using salt during cooking, only 82.6% reported doing so for foods prepared for their children. However, this proportion is notably higher compared to some previous research. For example, one study reported that only 46% of parents added salt during food preparation for their children (12). Similarly, while around 41.4% of parents reported adding salt at the table, less than a quarter (18%) reported their child’s use of salt at the table. These findings are broadly consistent with prior studies linking parent and child sodium intake and parental salt-related behaviors with children’s sodium excretion (18, 19). This discrepancy between parental and child salt use at the table may suggest that some parents are trying to limit their children’s salt intake, even if their own salt consumption remains high (17).

In unadjusted exploratory analyses, our study revealed various associations between salt-related knowledge and attitudes and specific salt-related behaviors. Specifically, parents who recognized the long-term health implications of excessive salt intake during childhood showed several favorable behaviors, including being less likely to place salt shakers on the table and more likely to limit fast food consumption, purchase foods labeled as “no added salt” or “reduced sodium,” and request meals without added salt when dining out (12). However, this knowledge was also associated with greater reported salt use when preparing food for their children. Parent-focused educational research has also shown that improving salt-related knowledge and attitudes can be accompanied by improvements in selected salt-reduction behaviors, including discretionary salt use and food-label practices (13). These findings underscore the importance of knowledge and attitudes in exploring parental behaviors related to salt consumption. Future interventions should therefore include educational strategies aimed at enhancing parental awareness of the long-term health consequences of high salt intake in children, while also addressing barriers to behavior change (15).

While our study indicated that parents’ awareness of excessive salt consumption among Saudi adults was not significantly associated with reduced discretionary salt use, these parents were more likely to use spices and herbs as salt alternatives and request meals prepared without added salt when dining out. Similarly, an Australian parent study found that parents with greater awareness of salt overconsumption were more inclined to purchase low-sodium foods (12). These findings highlight the need for educational campaigns emphasizing the health risks of high salt intake, particularly in the context of salt-related dietary practices and processed-food consumption in Saudi Arabia (9). However, it is important to acknowledge that awareness campaigns alone may not be sufficient to drive behavioral changes related to salt use (20). As such, interventions should consider a broader approach, including improving food literacy and addressing factors influencing food choices (21, 22).

While our study indicated that salt-related knowledge was associated with some positive salt-reduction behaviors, a significant proportion of parents who were aware of the negative health effects of high salt intake in children still added salt to food prepared for their child. As observed in other populations (12), our results suggest that knowledge alone is insufficient to explain behavior, indicating a need to further explore sociocultural factors unique to the Saudi setting. Previous studies suggest that environmental, demographic, cultural, educational, and economic factors, together with cooking skills, food availability, and taste preferences, can influence salt-related behaviors (13, 22). For instance, cultural norms around food preparation and taste preferences developed in childhood may influence salt consumption (17, 23). Therefore, future research and interventions should adopt a multifaceted approach, considering the interplay of these various influences on parental salt-related behaviors.

While our findings indicated that a majority of parents recognized the importance of limiting their child’s salt intake, maintaining this awareness and positive attitude through continued salt-reduction campaigns remains important. This is because such attitudes have been linked to positive salt-related behaviors, including a decreased likelihood of placing salt shakers on tables, reduced frequency of children adding salt to food, increased avoidance of pre-packaged and fast foods, a preference for foods labeled as “no added salt” or “reduced sodium,” and proactive requests for meals prepared without salt when dining out (12). These observations align with broader consumer-behavior research showing that concern about salt and motivation to reduce intake may encourage greater attention to food labels (24). Therefore, alongside enhancing knowledge, improving parental attitudes toward dietary salt should be a key consideration in future salt-reduction strategies. As Grimes et al. reported, consumer awareness campaigns targeting parents may lead to modest improvements in recognition of the importance of limiting children’s salt intake and some discretionary salt-use behaviors, even if broader changes in salt-related knowledge, attitudes, and behaviors are limited (15).

After adjustment for potential confounders and correction for multiple testing across the primary KAP coefficients, support for stronger action on salt in child-targeted foods was associated with higher odds of not placing a salt shaker on the table (aOR = 3.80, 95% CI 1.65–8.79, raw *p* = 0.002, *q* = 0.032); however, because this protective outcome was common and the confidence interval was wide, the estimate had limited precision and should be interpreted cautiously. This association suggests that parents who support broader salt-reduction initiatives may also be more likely to adopt healthier practices within the home food environment. Similar findings have been reported in previous studies, where greater parental awareness and positive attitudes toward salt reduction were associated with lower discretionary salt use and other recommended household practices (15). Furthermore, parental salt-related behaviors have been associated with children’s sodium intake, with the presence and use of table salt associated with higher 24-h urinary sodium excretion among children (19). Together, these findings highlight the important role of parents in creating a home environment that supports healthy dietary habits.

In mutually adjusted models, the association observed before FDR correction, indicating that parents who believed Saudi children consume excessive salt had lower odds of requesting salt-free restaurant meals, did not remain statistically significant after false-discovery-rate correction (raw *p* = 0.035, *q* = 0.280). More broadly, this highlights the challenges parents encounter in translating nutritional awareness into healthier choices outside the home. Factors such as limited control over food preparation, uncertainty about the feasibility of modifying restaurant meals, social dining practices, and perceived inconvenience may reduce the likelihood of requesting low-salt options despite awareness of the health risks. Similar discrepancies between nutrition knowledge and dietary behavior have been described in previous studies, suggesting that awareness alone does not always lead to behavioral change, particularly when environmental and contextual barriers exist (15, 25).

Childhood taste preferences and parental feeding practices may also contribute to dietary behavior. Human studies suggest that early dietary experience and childhood development are associated with acceptance and preference for salty tastes (23, 26). Saltiness can also interact with other taste qualities, including bitterness, which may influence food acceptance (27). Separately, parental feeding practices such as pressure to eat can affect children’s food intake and affective responses (28). These findings support the possibility that early dietary exposure and feeding practices shape later eating preferences, although these mechanisms were not directly assessed in the present study.

Social and environmental determinants of dietary behavior further compound this gap. Saudi Arabia’s rapid modernization has significantly escalated the societal availability and continuous consumption of highly processed, packaged, and fast foods, which contain dense amounts of hidden sodium (29). Market-level implementation of low-sodium alternatives is severely hindered by systemic reliance on un-reformulated food imports and significant private industry resistance, which directly limit local retail availability and product variety. Furthermore, profound socioeconomic disparities restrict consumer purchasing power, rendering the high cost of healthy alternatives a significant structural barrier (30). Consequently, even highly motivated parents face immense environmental resistance when trying to operationalize their nutritional knowledge into practice. Future public health interventions must therefore move beyond individual-focused awareness campaigns, prioritizing structural shifts such as industrial sodium regulations, mandatory clear front-of-pack food packaging guidelines, and expanding the affordability and distribution of low-sodium options across the Kingdom.

### Strengths and limitations

The study possesses several distinct strengths that contribute valuable evidence to the public health nutrition literature. First, it addresses a highly relevant and pressing pediatric public health issue—excessive dietary sodium intake—within Saudi Arabia, where regional evidence and baseline empirical data remain relatively limited. Second, the study includes a reasonably large overall sample size (*n* = 655), providing a useful basis for descriptive analyses, although precision varied across outcome-specific models. Third, by simultaneously evaluating parental knowledge, attitudes, and dietary practices within the same cohort, this study uniquely allows for the direct identification and mapping of critical awareness-practice gaps. Consequently, these findings offer clear, actionable baseline implications for designing future national public health education campaigns and structural sodium-reduction interventions across the Kingdom.

Nonetheless, several structural limitations should be acknowledged. Due to its cross-sectional design, this study captures only a snapshot in time and cannot establish definitive causal relationships or directional pathways among parental characteristics, knowledge, and behaviors. Furthermore, because the survey was distributed as an online instrument via digital networks, it is subject to inherent selection bias and digital access bias. The recruitment methods relied on smartphone link sharing, which naturally favored individuals with reliable internet connectivity, higher digital literacy, and a baseline interest in health-focused topics.

Consequently, our sample’s demographic profile—characterized by a high proportion of female respondents (70.5%) and highly educated individuals (77.1% holding a tertiary degree)—may not fully reflect the broader Saudi parent population. Compared with the approximately balanced sex distribution among the Saudi population reported in the Saudi Census 2022 (50.2% male and 49.8% female) (31), women were over-represented in this convenience sample. This difference, together with the high proportion of university-educated respondents, suggests selection toward female and more highly educated caregivers and limits population-level generalizability. National census estimates from GASTAT are not directly parent-specific and are used here only as an external benchmark. Additionally, while the survey link was distributed broadly across public digital channels, geographical distribution metrics across specific provinces or rural versus urban divides were not explicitly tracked in the primary database, preventing any regional sub-group analyses. In addition, detailed child-specific characteristics, including children’s age distribution, sex, number of children per household, and child chronic illness status, were not collected, limiting the ability to conduct child-level subgroup analyses.

The survey instrument, while meticulously adapted from previous validated international frameworks and subjected to a forward-backward translation protocol with an independent pilot phase, lacks formal statistical psychometric validation (such as factor analysis or internal consistency reliability coefficients) within the specific cultural context of Saudi Arabia. Furthermore, because all data were self-reported, the findings are inherently vulnerable to recall bias and social desirability bias, where parents might overreport protective nutritional choices or underreport discretionary salt additions to align with perceived socially acceptable parenting standards.

Finally, although the overall sample comprised 655 caregivers, effective sample sizes and event counts varied across outcome-specific models. Some outcomes and predictor categories were imbalanced or sparse, producing wide confidence intervals and limited precision. The large number of exploratory comparisons also increased the risk of false-positive findings; multiplicity-adjusted and unadjusted results should therefore be interpreted cautiously and confirmed in adequately powered, probability-based studies.

## Conclusion

In this online convenience sample, this study provides baseline insights into the knowledge, attitudes, and discretionary practices of parents and caregivers in Saudi Arabia regarding children’s dietary salt intake. The findings reveal a generally high level of health awareness regarding the long-term clinical risks of excessive salt exposure, along with an expressed attitude prioritizing salt reduction in children’s diets compared to their own. However, a clear gap remains in parents’ understanding of actual pediatric consumption levels, and a notable knowledge–behavior gap is apparent in household discretionary salt practices.

After adjustment for potential confounders and correction for multiple testing across primary KAP coefficients, the adjusted analyses provided limited evidence of independent associations between parental KAP and the selected behaviors. Only one association—between support for stronger action on child-targeted foods and lower odds of keeping a salt shaker on the table—survived FDR correction; however, its wide confidence interval and common outcome frequency warrant cautious interpretation and replication.

These observed statistical associations provide a valuable foundation for future public health frameworks. Future research utilizing longitudinal designs and objective clinical measures, such as 24-h urinary sodium excretion, is required to build upon these baseline data and guide the development of targeted, structurally supportive public health strategies and industrial sodium-reduction interventions across Saudi Arabia.

## Data Availability

The raw data supporting the conclusions of this article will be made available by the authors, without undue reservation.
